# Angiographic characteristics of older patients with non-ST elevation myocardial infarction undergoing invasive strategy: A pre-specified analysis from the SENIOR-RITA trial

**DOI:** 10.1093/ehjopen/oeag131

**Published:** 2026-08-20

**Authors:** Graziella Pompei, Francesca Rubino, Kenny Jenkins, Hanna Ratcovich, David Austin, Shrilla Banerjee, Gill Louise Buchanan, Shahid Junejo, Abdul Nasser, Nehal Hussein, Jolanta Sobolewska, Timothy Williams, Stuart Hood, Awsan Noman, Akhlaque Uddin, Manas Sinha, Robert Butler, John Irving, Robert F Storey, David E Newby, Keith A A Fox, Stuart Pocock, Vijay Kunadian

**Affiliations:** Translational and Clinical Research Institute, Faculty of Medical Sciences, Newcastle University, 4th Floor William Leech Building, Newcastle upon Tyne NE2 4HH, UK; Translational and Clinical Research Institute, Faculty of Medical Sciences, Newcastle University, 4th Floor William Leech Building, Newcastle upon Tyne NE2 4HH, UK; Translational and Clinical Research Institute, Faculty of Medical Sciences, Newcastle University, 4th Floor William Leech Building, Newcastle upon Tyne NE2 4HH, UK; Translational and Clinical Research Institute, Faculty of Medical Sciences, Newcastle University, 4th Floor William Leech Building, Newcastle upon Tyne NE2 4HH, UK; Academic Cardiovascular Unit, The James Cook University Hospital, South Tees NHS Trust, Marton Road, Middlesbrough TS4 3BW, UK; Population Health Science Institute, Faculty of Medical Sciences, Newcastle University, Baddiley-Clark Bldg, Newcastle upon Tyne NE2 4AX, UK; Department of Cardiology, Surrey and Sussex Healthcare NHS Trust, Canada Ave, Redhill RH1 5RH, UK; Cardiology Department, North Cumbria Integrated Care National Health Service Foundation Trust, Infirmary Street, Carlisle CA2 7HY, UK; Departments of Endocrinology and Cardiology, South Tyneside and Sunderland NHS Foundation Trust, Kayll Road, Sunderland SR4 7TP, UK; Department of Cardiology, South Tyneside and Sunderland NHS Foundation Trust, Harton Lane, South Shields NE34 0PL, UK; Cardiology Unit, Salford Royal NHS Foundation Trust, Stott Ln, Salford M6 8HD, UK; Department of Cardiology, Royal Oldham Hospital, Rochdale Rd, Oldham OL1 2JH, UK; Department of Cardiology, Maidstone and Tunbridge Wells NHS Trust, Hermitage Lane, Maidstone, Kent ME16 9QQ, UK; Cardiology Department, Royal Alexandra Hospital, Paisley PA2 9PN, Lanarkshire, UK; University of Aberdeen, University Office, King's College, Aberdeen AB24 3FX, UK; Cardiology Department, Nottingham University Hospitals NHS Trust, Hucknall Road, Nottingham NG51PB, UK; Cardiology Unit, Salisbury NHS Foundation Trust, Odstock Road, Salisbury SP2 8BJ, UK; Cardiology Unit, Royal Stoke University Hospital, Newcastle Road, Stoke-on-Trent ST4 6QG, UK; Cardiology Department, Ninewells Hospital, Ninewells Avenue, Dundee DD1 9SY, UK; Division of Clinical Medicine, University of Sheffield, Beech Hill Road, Sheffield S10 2RX, UK; NIHR Sheffield Biomedical Research Centre, Sheffield Teaching Hospital NHS Foundation Trust, Glossop Road, Sheffield S10 2JF, UK; BHF Centre for Cardiovascular Science, University of Edinburgh, Chancellor's Building, 49 Little France Crescent, Edinburgh EH16 4SB, UK; BHF Centre for Cardiovascular Science, University of Edinburgh, Chancellor's Building, 49 Little France Crescent, Edinburgh EH16 4SB, UK; Medical Statistics Department, London School of Hygiene and Tropical Medicine, Keppel Street, London WC1E 7HT, UK; Translational and Clinical Research Institute, Faculty of Medical Sciences, Newcastle University, 4th Floor William Leech Building, Newcastle upon Tyne NE2 4HH, UK; Cardiothoracic Centre, Freeman Hospital, Newcastle upon Tyne Hospitals NHS Foundation Trust, Freeman Road, High Heaton, Newcastle upon Tyne NE7 7DN, UK

**Keywords:** Non-ST-elevation acute coronary syndrome, Invasive strategy, Conservative strategy, Older patients, Coronary angiography, Percutaneous coronary intervention

## Abstract

**Aims:**

In the SENIOR-RITA trial, among older adults with non-ST-elevation myocardial infarction (NSTEMI), an invasive strategy did not reduce the composite outcome of cardiovascular death or non-fatal myocardial infarction compared with a conservative strategy over a median follow-up of 4.1 years.

**Objectives:**

To describe the angiographic characteristics of patients randomized to the invasive arm.

**Methods and results:**

SENIOR-RITA was a prospective, multicentre, randomized controlled trial recruiting NSTEMI patients aged ≥75 years. From November 2016 to March 2023, 1518 patients from 48 UK sites were randomized 1:1 to an invasive or conservative strategy. Procedural data were collected, and angiographic data were analysed using quantitative coronary angiography at the Newcastle University angiographic core laboratory using QAngio® XA Medis. Of 680 patients undergoing angiography, quantitative coronary angiography analysis was performed in 667 participants. Obstructive coronary artery disease was found in 76.8%, and 13% had prior coronary surgical revascularization. A total of 1173 lesions were identified. Most lesions (29.5%) were classified as type B2 according to the ACC/AHA classification. Lesions in patients not receiving percutaneous coronary intervention (PCI) were angiographically more complex than those treated with PCI, with a higher prevalence of type C lesions, bifurcation, ostial lesions, and the presence of collaterals. Overall, 330 patients (49.5%) underwent PCI, which was successful in 96.5% with minimal complications.

**Conclusion:**

The SENIOR-RITA angiography sub-study is the largest subgroup analysis to date in older NSTEMI adults, providing important insights into the angiographic characteristics and emphasizing the importance of a holistic, patient-centred care for older adults.

## Graphical Abstract

**Graphical Abstract:**
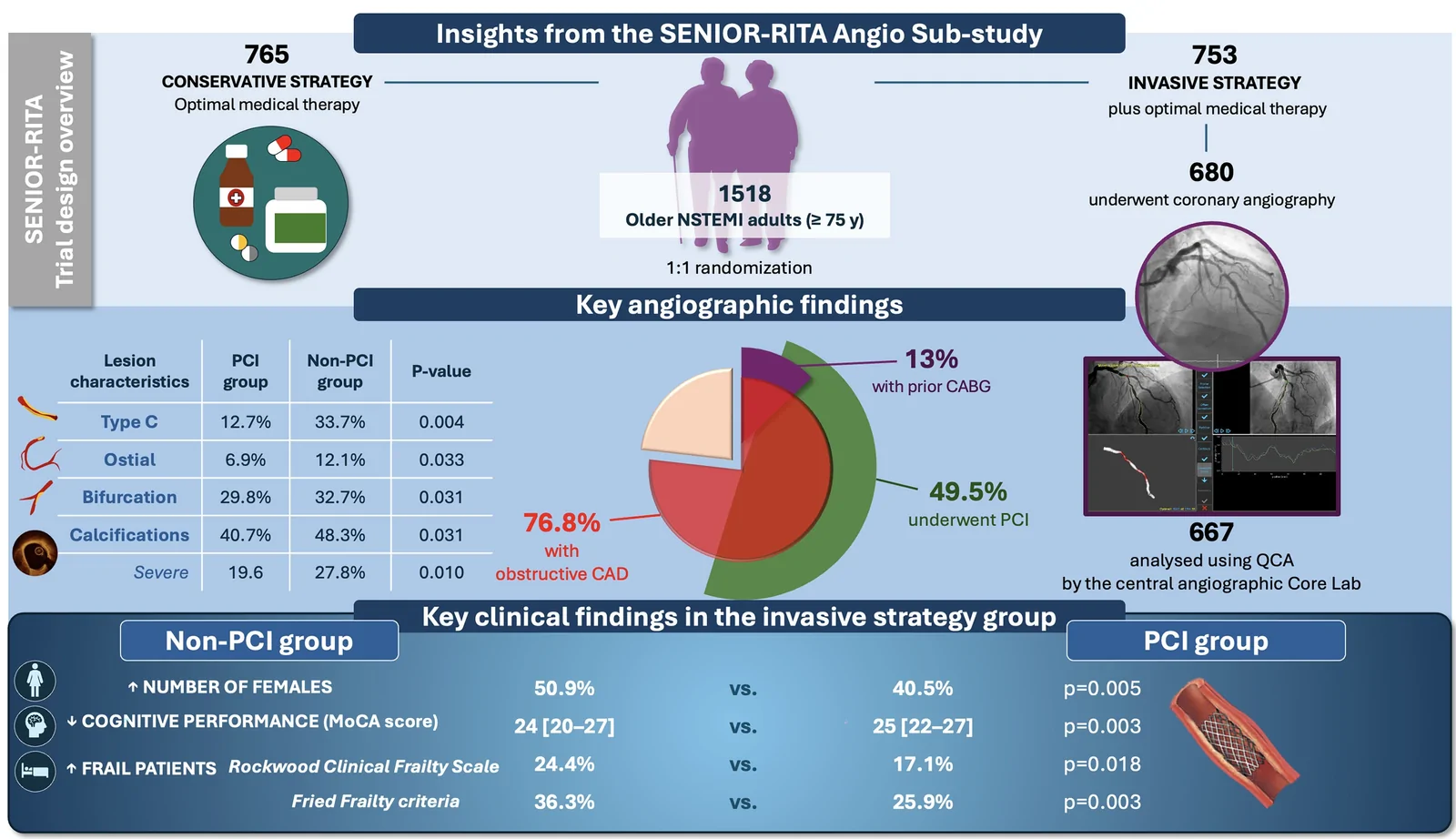
**Key angiographic and clinical findings from the SENIOR-RITA Angio Sub-study.** Abbreviations. CABG, coronary artery bypass grafting; CAD, coronary artery disease; MoCA, Montreal Cognitive Assessment; NSTEMI, non-ST-segment elevation myocardial infarction; PCI, percutaneous coronary intervention; QCA, quantitative coronary angiography.

## Introduction

The recently published British Heart Foundation SENIOR-RITA (Older patients with non-ST SEgmeNt elevation myocardial infarction Randomized Interventional TreAtment) trial enrolled older non-ST-elevation myocardial infarction (NSTEMI) patients aged ≥75 years. Patients were randomly allocated to conservative management, consisting of optimal medical therapy, or an invasive strategy, consisting of coronary angiography in addition to optimal medical therapy and revascularization if needed.^1^ Among older adults with NSTEMI, an invasive strategy did not reduce the composite outcome of cardiovascular death or non-fatal myocardial infarction (MI) compared with a conservative strategy over a median follow-up of 4.1 years. MIs and subsequent revascularizations were lower in patients managed by an invasive strategy compared with those managed conservatively.

In a previous angiographic analysis from a prospective cohort study (ICON1), we showed that frail older adults presenting with non-ST-elevation acute coronary syndrome have more adverse baseline angiographic characteristics, which makes invasive management more challenging.^2–4^ Frailty is strongly associated with severe culprit lesion calcifications and thin cap fibroatheroma.^2,3^ Given that older adults are heterogeneous, with conditions such as frailty, cognitive impairment, and comorbidity impacting clinical outcomes, detailed angiographic analysis is required to better understand the SENIOR-RITA trial findings and its implications for clinical practice. A detailed angiographic analysis could help elucidate how these factors influence response to treatments or the clinical decision-making in this population.

In the SENIOR-RITA trial, for patients randomized to the invasive strategy who underwent coronary angiography, the angiographic projections for the purpose of quantitative coronary angiography (QCA) computation were prospectively collected. The aim of this pre-specified study is to describe the angiographic characteristics of older patients randomized to the invasive arm of the SENIOR-RITA trial.

## Methods

The design and methods of the SENIOR-RITA trial have been published previously.^1^ Briefly, the SENIOR-RITA trial was a prospective, multicentre, open-label, randomized controlled trial that recruited patients ≥75 years of age with NSTEMI to test an invasive vs. a conservative treatment strategy. All patients provided informed consent. A consultee declaration was obtained from a family member or a caregiver for patients with cognitive impairment. The trial was funded by the British Heart Foundation (CS/15/7/31679) and sponsored by Newcastle upon Tyne Hospitals NHS Foundation Trust (Sponsor reference 7910). The Newcastle Clinical Trials Unit (UKCRC registration ID 22) managed and coordinated the trial. The protocol was approved by the UK Health Research Authority (16/NE/0238). This subgroup analysis was pre-specified in the trial protocol and statistical analysis plan, which is available along with the full text of the SENIOR-RITA trial manuscript.^1^

## Trial population

Patients with a clinical diagnosis of NSTEMI aged ≥75 years were enrolled. Exclusion criteria included diagnosis of ST-elevation myocardial infarction, unstable angina, cardiogenic shock, or life expectancy of less than 1 year. Detailed inclusion and exclusion criteria are described in the Supplementary Material online, *Table S1*. Cognitive impairment was evaluated using the Montreal Cognitive Assessment (MoCA) score from 0 to 30, with a score ≥26 indicating normal cognitive function.

## Randomization and treatment

All patients were randomly assigned in a 1:1 ratio to an invasive treatment with revascularization if appropriate, plus optimal medical therapy (the invasive strategy group), or to conservative management with optimal medical therapy alone (conservative-strategy group). Randomization was on a 1:1 basis using a variable-length block stratified method with randomly selected block sizes of two, four, six, and eight. Randomization was performed at each site using a secure web-based system.

Optimal medical therapy included aspirin (75 mg daily), a P2Y_12_ receptor antagonist, a beta-blocker (target heart rate of 60 to 70 beats per minute), statin therapy, and an angiotensin-converting enzyme inhibitor or angiotensin II receptor blocker. Comorbidities such as hypertension, diabetes, and hypercholesterolaemia were managed according to the pertinent clinical practice guidelines.

Patients assigned to the invasive group underwent coronary angiography with coronary revascularization if appropriate and received optimal medical therapy. Coronary angiography was performed according to local guidelines. Coronary revascularization was carried out within 3 to 7 days, when appropriate, through either percutaneous coronary intervention (PCI) or coronary artery bypass grafting (CABG), as determined by the attending cardiologist and the multidisciplinary team. In the conservative strategy group, coronary angiography was permitted in cases of clinical deterioration when deemed necessary by the treating physicians.

## Quantitative coronary angiography

For the present analysis, operators were required to acquire prospectively angiographic projections of all coronary arteries in patients allocated to the invasive strategy and undergoing coronary angiography. QCA computation of lesions was performed off-line by the central angiographic core lab (Newcastle University) using QAngioXA/3D software in agreement with the step-by-step procedure validated in previous studies.^5^ All the core laboratory reviewers (GP, FR, DS, DE, HR, and KJ) were certified operators blinded to all other data. To mitigate inter-observer variability, the reviewers independently conducted the angiographic analyses. A second reviewer then assessed the analyses and provided corrections where necessary, and a third supervisor (VK) validated all approved results and corrections to ensure accuracy and consistency.

Quantitative angiographic analysis was performed on the angiographic frame with maximal stenosis, with hand-drawn contours around the lumen diameter and lesion length at the site of each diseased vessel. The analysis was conducted according to standard criteria and definitions as previously defined.^6–9^ This included lesion quantitative analysis of lesion diameter stenosis, length, reference vessel diameter, lesion area, and minimum lumen diameter. Obstructive coronary artery disease (CAD) was defined as a diameter stenosis greater than 50% as assessed by QCA. Qualitative analysis was used to categorize calcification grade, eccentric, diffuse, ostial or bifurcation lesion, ulcer, pulsatile or slow flow, collaterals and collateral score (scored between 0 and 3), thrombus grade (score between 0 and 5), and lesion complexity (defined as type A, type B1, type B2, or type C according to the American College of Cardiology/American Heart Association (ACC/AHA) classification). Lesion calcifications were defined as (i) mild (faint radiopacities noted during the cardiac cycle), (ii) moderate (dense radiopacities), and (iii) severe (dense radiopacities noted without cardiac motion before contrast injection, generally compromising both sides of the arterial lumen).

## Statistical analysis

Categorical variables were reported as numbers and percentages (*n*, %) and analysed with the *χ*^2^ and Monte Carlo tests. Continuous variables were assessed for normal distribution using the Shapiro-Wilk test and Q–Q plots and then displayed as either mean (standard deviation) for normal data or median [interquartile range (IQR)] for non-normally distributed data. Normally distributed continuous variables were compared using an independent *t*-test, while the Mann–Whitney U test with Monte Carlo was used for non-normally distributed data. All statistical analyses were performed using the Statistical Package for the Social Sciences (SPSS version 29.0.1.1, IBM Corporation). Significance was predetermined at the level of two-tailed *P* < 0.05.

## Results

**Figure 1:**
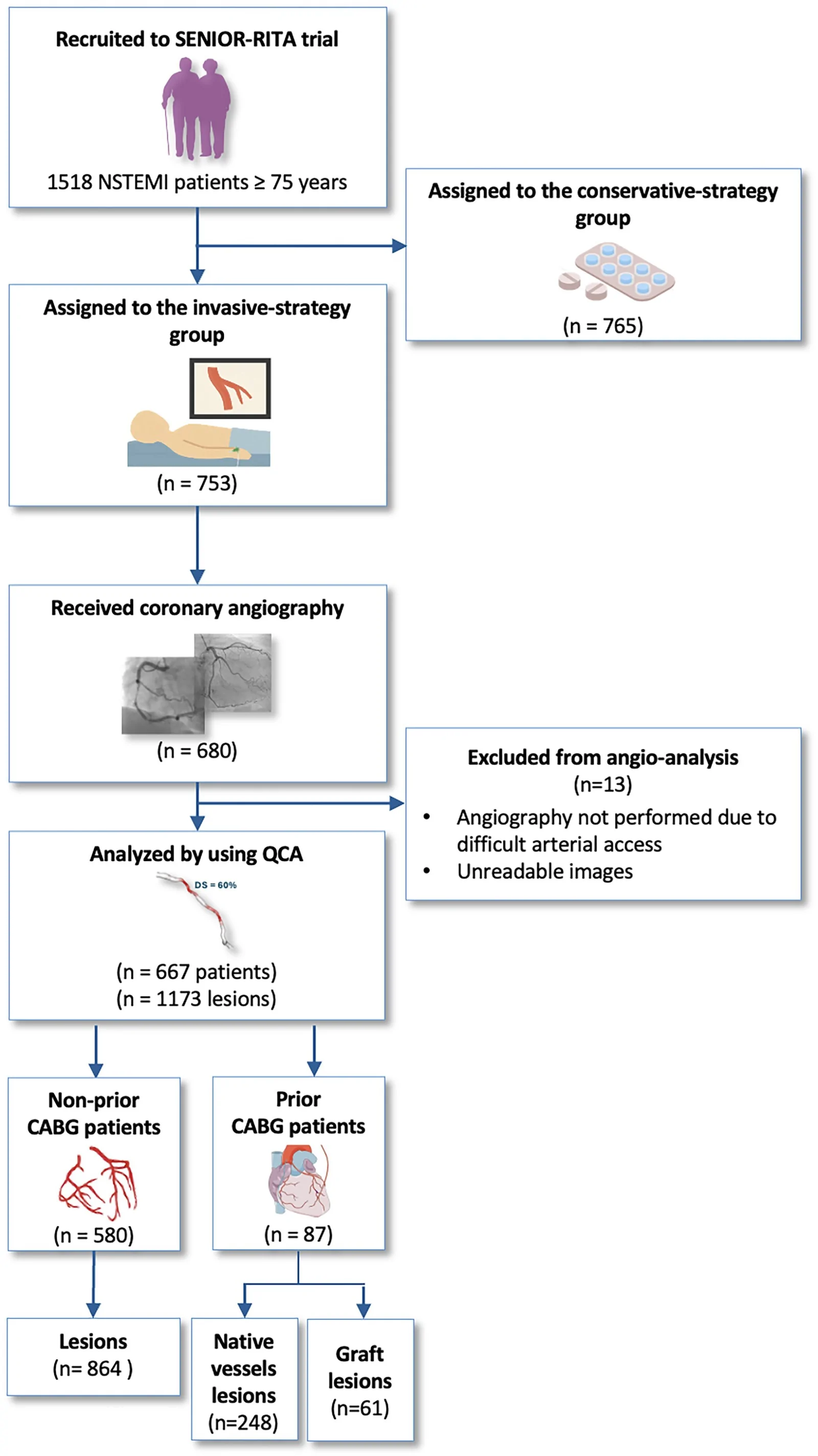
SENIOR-RITA Angio Sub-study flow chart. Figure 1 shows the study flow chart of patients included in the angiographic analysis. Abbreviations. CABG, coronary artery bypass grafting; QCA, quantitative coronary angiography.

Out of the 680 patients who received coronary angiography in the invasive strategy group, a total of 667 were analysed. The main reasons for excluding angiograms from analysis were as follows: coronary angiography was not performed due to difficult arterial access and unreadable images (*Figure 1*). Eighty-seven patients (13%) had previously undergone CABG surgery. Out of the 667 analysed patients, 512 (76.8%) had obstructive CAD. The total number of lesions was 1173, of which 1112 were on native vessels and 61 on grafts. The most common location was the left anterior descending (LAD) artery (35%), followed by the right coronary artery (RCA) (29.9%). A total of 255 (21.7%) lesions were occluded, and the majority (29.5%) were type B2 according to the ACC/AHA classification. Bifurcation involvement was present in 31.5% of lesions, ostial lesions in 10.1%, and diffuse disease (defined as lesion length ≥ 20 mm) in 22.3%.

### Baseline clinical characteristics of patients undergoing invasive strategy

Among the 753 patients allocated to receive an invasive strategy, patients who did not receive PCI were more frequently female (50.9% vs. 40.5%, *P* = 0.005) and had poorer cognitive performance, with a lower median MoCA score [24 (IQR 20–27) vs. 25 (IQR 22–27), *P* = 0.003]. A higher proportion of patients not treated with PCI were classified as frail according to both the Rockwood Clinical Frailty Scale (24.4% vs. 17.1%, *P* = 0.018) and the Fried Frailty criteria (36.3% vs. 25.9%, *P* = 0.003). Additionally, hypertension (68.7% vs. 61.5%, *P* = 0.043) and peripheral vascular disease (9.8% vs. 5.1%, *P* = 0.017) were more prevalent among individuals who did not undergo PCI. There were no statistically significant differences in age, Charlson comorbidity index, or the prevalence of other cardiovascular or chronic conditions between the two groups (*Table 1*). There was no difference in discharge medical therapy received by patients undergoing invasive and conservative care, as shown in Supplementary material online, *Table S2*, meaning all patients were treated according to guideline-recommended secondary prevention therapy.

**Table 1:** Baseline demographic and clinical characteristics of patients undergoing invasive strategy

Characteristics	Total patients who had invasive strategy (*N* = 753)	Patients who had PCI (*N* = 351)	Patients who did not have PCI^a^ (*N* = 377)	*P* value
Female sex, no. (%)	337 (44.8)	142 (40.5)	192 (50.9)	0.005
Age, yr (SD)	82.5 ± 4.7	82.7 ± 4.8	82.6 ± 4.7	0.998
≥75 to <80, no. (%)	211 (28)	96 (27.4)	101 (26.8)	0.868
≥80 to <85, no. (%)	304 (40.4)	140 (39.9)	154 (40.8)	0.821
≥85 to <90, no. (%)	182 (24.2)	85 (24.2)	96 (25.5)	0.732
≥90 to <95, no. (%)	47 (6.2)	27 (7.7)	20 (5.3)	0.227
≥95, no. (%)	9 (1.2)	3 (0.9)	6 (1.6)	0.508
Median MoCA [IQR]	25 [21, 27]	25 [22, 27]	24 [20, 27]	0.003
Impaired (MoCA < 26), no./total no. (%)	433/724 (59.8)	190 (54.1)	232 (61.5)	0.051
Median Rockwood Frailty score [IQR]	3 [2, 4]	3 [2, 4]	3 [3, 4]	<0.001
Frail (Rockwood score ≥ 5), no. (%)	153/753 (20.3)	60 (17.1)	92 (24.4)	0.018
Very fit (1)	103/752 (13.7)	59 (16.8)	41 (10.9)	0.023
Well, without active disease (2)	134/752 (17.8)	74 (21.1)	52 (13.8)	0.011
Well, with treated comorbidities (3)	198/752 (26.3)	89 (25.4)	102 (27.1)	0.614
Apparently vulnerable (4)	165/752 (21.9)	69 (19.7)	89 (23.6)	0.209
Mildly frail (5)	97/752 (12.9)	40 (11.4)	57 (15.1)	0.156
Moderately frail (6)	47/752 (6.2)	17 (4.8)	30 (8)	0.098
Severely frail (7)	8/752 (1.1)	3 (0.9)	5 (1.3)	0.726
Median Fried Frailty score [IQR]	2 [1, 3]	1 [1, 3]	2 [1, 3]	0.001
Robust (0), no. (%)	150/716 (20.9)	80 (22.8)	63 (16.7)	0.040
Pre-frail (1 or 2), no. (%)	335/716 (46.8)	166 (47.3)	157 (41.6)	0.136
Frail (≥3), no. (%)	231/716 (32.3)	91 (25.9)	137 (36.3)	0.003
Median Charlson age-adjusted comorbidity index [IQR]	5 [4, 6]	5 [4, 6]	5 [4, 6]	0.229
**Smoking status**				
Current smoker, no./total no. (%)	35/748 (4.7)	22 (6.3)	12 (3.2)	0.054
Former smoker, no./total no. (%)	358/748 (47.9)	168 (47.9)	178 (47.2)	0.882
Never smoked, no./total no. (%)	355/748 (47.5)	159 (45.3)	184 (48.8)	0.373
Hypertension, no./total no. (%)	490/753 (65.1)	216 (61.5)	259 (68.7)	0.043
Diabetes, no./total no. (%)	232/753 (30.8)	104 (29.6)	119 (31.6)	0.575
Hypercholesterolaemia, no./total no. (%)	242/752 (32.2)	113 (32.2)	119 (31.6)	0.874
History of renal disease, no./total no. (%)	156/753 (20.7)	68 (19.4)	85 (22.6)	0.317
Previous MI, no./total no. (%)	247/753 (32.8)	109 (31.1)	134 (35.5)	0.209
Previous PCI, no./total no. (%)	163/752 (21.7)	78 (22.2)	82 (21.8)	0.929
Previous CABG, no./total no. (%)	101/753 (13.4)	41 (11.7)	60 (15.9)	0.108
History of peripheral vascular disease, no. (%)	57/753 (7.6)	18 (5.1)	37 (9.8)	0.017
History of TIA/stroke, no./total no. (%)	128/753 (17)	55 (15.7)	70 (18.6)	0.326
History of COPD, no./total no. (%)	115/753 (15.3)	55 (15.7)	56 (14.9)	0.837
History of congestive heart failure, no./total no. (%)	73/753 (9.7)	28 (8)	45 (11.9)	0.084
Days from admission to randomization [IQR]	2 [1, 3]	2 [1, 3]	2 [1, 3]	

^a^The analyses reported in the table exclude individuals who received CABG post-randomization (*n* = 25)

**Abbreviations.** CABG, coronary artery bypass graft; COPD, chronic obstructive pulmonary disease; IQR, interquartile range; MI, myocardial infarction; MoCA, Montreal Cognitive Assessment; PCI, percutaneous coronary intervention; SD, standard deviation; TIA, transient ischaemic attack

### Angiographic characteristics of patients who did and did not have percutaneous coronary intervention

**Figure 2:**
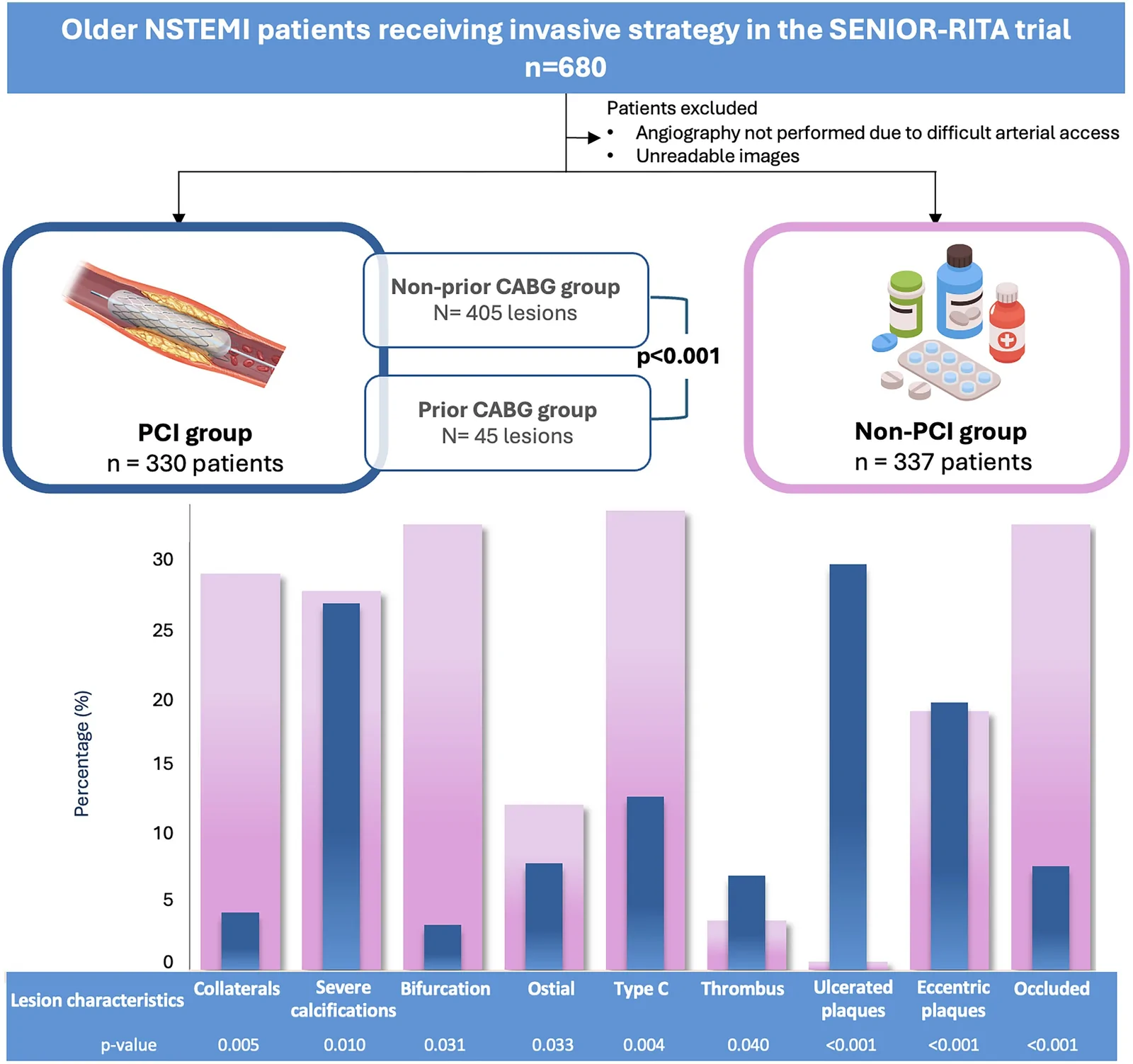
Lesion characteristics in PCI and non-PCI group. Figure 2 shows the lesion characteristics of patients in the PCI and the non-PCI groups. Percentages are calculated on the total number of analysed lesions per each group (PCI and non-PCI performed).

Among the 512 patients with obstructive CAD, 330 (64.5%) underwent PCI. Significant differences were observed between patients receiving PCI and those who did not have PCI, particularly in the number of diseased vessels and lesion characteristics (*Table 2* and *Figure 2*).

**Table 2:** Baseline angiographic characteristics of patients allocated to the invasive strategy group

Angiographic characteristics	All analysed patients *N* = 667	Patients who had PCI *N* = 330	Patients who did not have PCI *N* = 337	*P* value
**Patients with diseased vessels, no. (%)**	512 (76.8)	330 (100)	183 (54.3)	<0.001
**Total number of lesions, no.**	1173	710	463	
Lesions on native vessels, no. (%)	1112 (94.8)	300 (42.3)	165 (35.6)	< 0.001
Lesions on grafts, no. (%)	61 (5.2)	29 (4.1)	18 (3.9)	
**Dominance, no. (%)**				
Right	573 (85.9)	289 (87.6)	284 (84.3)	0.270
Left	64 (9.6)	29 (8.8)	35 (10.4)	
Co-dominant	30 (4.5)	11 (3.3)	19 (5.6)	
**Number of diseased native vessels, no. (%)**	932			
0	155 (23.2)	0 (0)	155 (46)	<0.001
1	217 (32.5)	156 (47.3)	61 (18.1)	
2	170 (25.5)	110 (33.3)	60 (17.8)	
3	125 (18.7)	63 (19.1)	62 (18.4)	
**Total number of lesions on native vessels, no.** (% of the analysed patients)	1112			
0	155 (23.2)	0 (0)	155 (46)	<0.001
1	177 (26.5)	126 (38.2)	51(15.1)	
2	148 (22.2)	99 (30.3)	49 (14.5)	
≥3	187 (28.0)	104 (31.5)	83 (24.6)	
**Lesion location, no.** (%, calculated on the total number of lesions)		**Lesions treated with PCI** ***N*** **=** **450**	**Lesions not treated with PCI** ***N*** **=** **723**	
LAD artery	366 (31.2)	163 (36.2)	203 (28.1)	0.305
LCx artery	206 (17.6)	75 (16.7)	131 (18.1)	
RCA	338 (28.8)	125 (27.8)	213 (29.5)	
Intermediate	14 (1.2)	2 (0.5)	12 (1.7)	
Diagonal	44 (3.8)	13 (2.9)	31 (4.3)	
Obtuse marginal	84 (7.2)	25 (5.6)	59 (8.2)	
PLA	18 (1.5)	6 (1.3)	12 (1.7)	
PDA from RCA	13 (1.1)	6 (1.3)	7 (1.0)	
LM	28 (2.4)	12 (2.7)	16 (2.2)	
PDA from LCx	1 (0.1)	1 (0.2)	0 (0.0)	
Graft to LAD	8 (0.7)	1 (0.2)	7 (1.0)	
Graft to LCx	28 (2.4)	14 (3.1)	14 (1.9)	
Graft to RCA	25 (2.1)	7 (1.6)	18 (2.5)	
**Lesions characteristics, no.** (%, calculated on the total number of lesions on both grafts and native vessels)				
Total occlusion	255 (21.7)	19 (4.2)	236 (32.7)	<0.001
Eccentric plaque	258 (22)	121 (26.9)	137 (19)	<0.001
**Calcifications**	532 (45.4)	183 (40.7)	349 (48.3)	0.031
Mild	104 (8.9)	50 (11.1)	54 (7.5)	0.010
Moderate	139 (11.9)	45 (10)	94 (13)	
Severe	289 (24.6)	88 (19.6)	201 (27.8)	
Diffuse disease	262 (22.3)	53 (11.8)	209 (29)	<0.001
Ostial	118 (10.1)	31 (6.9)	87 (12.1)	0.033
Bifurcation lesion	370 (31.5)	134 (29.8)	236 (32.7)	0.031
**Medina classification**				
0,0,1	40 (3.4)	6 (1.3)	34 (4.7)	0.077
0,1,0	73 (6.2)	30 (6.7)	43 (6)	
0,1,1	20 (1.7)	8 (1.8)	12 (1.7)	
1,0,0	31 (2.6)	21 (4.7)	10 (1.4)	
1,0,1	12 (1.0)	5 (1.1)	7 (1)	
1,1,0	66 (5.6)	31 (6.9)	35 (4.9)	
1,1,1	127 (10.8)	32 (7.1)	95 (13.2)	
Flow deceleration	48 (4.1)	25 (5.6)	23 (3.2)	0.027
Pulsatile flow	9 (0.8)	6 (1.3)	3 (0.4)	0.015
Slow flow	34 (2.9)	18 (4)	16 (2.2)	0.033
Collaterals	244 (20.8)	34 (7.6)	210 (29.1)	0.005
**Collaterals score**				
1	42 (3.58)	10 (2.2)	32 (4.4)	0.004
2	202 (17.2)	24 (5.3)	178 (24.7)	
**Thrombus**	61 (5.2)	35 (7.8)	26 (3.6)	0.040
**Thrombus grade**				
1	4 (0.3)	2 (0.4)	2 (0.3)	0.137
2	2 (0.2)	2 (0.4)	0 (0.0)	
3	17 (1.5)	11 (2.4)	6 (0.8)	
4	8 (0.7)	7 (1.6)	1 (0.1)	
5	29 (2.5)	13 (2.9)	16 (2.2)	
Ulcer	19 (1.6)	15 (3.3)	4 (0.6)	<0.001
Distal embolization	6 (0.5)	5 (1.1)	1 (0.1)	0.001
**Lesion complexity (ACC/AHA)**				
A	134 (11.4)	63 (14)	71 (9.8)	0.004
B1	316 (26.9)	141 (31.3)	175 (24.2)	
B2	346 (29.5)	167 (37.1)	178 (24.7)	
C	300 (25.6)	57 (12.7)	243 (33.7)	
**Pre-PCI angiographic measurements calculated in all the lesions (on both native vessels and grafts)**				
LL pre-PCI (mm), median [IQR]	9.7 [6.9, 14.4]	9.8 [6.9, 14]	9.6 [6.8, 14.6]	0.692
Lesions RVD (mm), median [IQR]	2.7 [2.2, 3.3]	2.7 [2.2, 3.3]	2.6 [2.1, 3.2]	0.040
Area stenosis pre-PCI (%), median [IQR]	92.6 [84.9, 98.7]	91.7 [84.9, 96.1]	93.4 [85, 100]	<0.001
DS pre-PCI (%), median [IQR]	73 [61.4, 89.5]	71.5 [61.5, 80.8]	74.9 [61.3, 100]	<0.001
MLD pre-PCI (mm), median [IQR]	0.7 [0.3, 1]	0.8 [0.5, 1.1]	0.6 [0.00, 1.0]	<0.001

**Abbreviations.** ACC/AHA, American College of Cardiology/American Heart Association; DS, diameter stenosis; IQR, interquartile range; LAD, left anterior descending; LCx, left circumflex; LL, lesion length; LM, left main; MLD, minimal lumen diameter; RCA, right coronary artery; PCI, percutaneous coronary intervention; PDA, posterior-descending artery; PLA, posterior-lateral artery; RVD, reference vessel diameter.

The PCI group had lesions that were less often totally occluded (4.2% vs. 32.7%, *P* < 0.001) and more frequently consisted of eccentric plaques (26.9% vs. 19%, *P* < 0.001), ulcerated plaques (3.3% vs. 0.6%, *P* < 0.001), and thrombus (7.8% vs. 3.6%, *P* = 0.040) compared with the non-PCI group.

The non-PCI group compared with the PCI group had more complex disease with a significantly greater proportion classified as type C lesions (33.7% vs. 12.7%, *P* = 0.004) and higher prevalence of ostial lesions (12.1% vs. 6.9%, *P* = 0.033), bifurcation lesions (32.7% vs. 29.8%, *P* = 0.031), and calcifications (48.3% vs. 40.7%, *P* = 0.031), most of which were severe (27.8% vs. 19.6%, *P* = 0.010). Collateral circulation was identified more frequently in the non-PCI group (29.1% vs. 7.6%, *P* = 0.005) (*Table 2*).

### Angiographic characteristics of patients without and with prior coronary artery bypass grafting

Among the 580 older NSTEMI patients without prior CABG, 425 (73.3%) presented with obstructive CAD at coronary angiography. The most common lesion location was the LAD artery (35% of lesions). Detailed angiographic characteristics of both culprit and non-culprit lesions are shown in Supplementary material online, *Tables S3* and *S4*.

Eighty-seven patients had previously undergone CABG surgery, and the majority of these patients had three-vessel CAD (70.1% vs. 11% in the non-prior CABG group, *P* < 0.001) (*Table 3*). Among the prior CABG group, a total of 309 lesions were reported (248 located on native vessels, while 61 on grafts). Pre-procedural Thrombolysis in Myocardial Infarction (TIMI) flow grade 0 in the LAD artery was observed in more patients with prior CABG (64.4% vs. 2.8% in the non-CABG group, *P* < 0.001). A similar significant difference was noted for the left circumflex (LCx) and RCA (see Supplementary material online, *Tables S5*, *S6*, and *S7*).

**Table 3:** Lesions characteristics (culprit and non-culprit lesions) in non-CABG patients and CABG patients

Patients, no.	Non-CABG patients *N* = 580	CABG patients *N* = 87	*P* value
**Dominance, no. (%)**			
Right	494 (85.2)	79 (90.8)	0.334
Left	58 (10.0)	6 (6.9)	
Co-dominant	28 (4.8)	2 (2.3)	
**Patients with diseased vessels, no. (%)**	425 (73.3)	87 (100)	<0.001
**Number of diseased vessels, no. (%)**	700	232	<0.001
0	155 (26.7)	0 (0)	
1	214 (36.9)	3 (3.5)	
2	147 (25.4)	23 (26.4)	
3	64 (11)	61 (70.1)	
**Patients receiving PCI, no. (%)**	293 (50.5)	37 (42.5)	0.206
**Total number of lesions, no.**	864	309	
**Lesion location no. (%)**			
LAD artery	288 (33.3)	78 (25.2)	<0.001
LCx artery	153 (17.7)	53 (17.2)	
RCA	264 (30.6)	74 (24)	
Intermediate	12 (1.4)	2 (0.7)	
Diagonal	43 (5)	1 (0.3)	
Obtuse marginal	59 (6.8)	25 (8.1)	
PLA	16 (1.9)	2 (0.7)	
PDA	12 (1.4)	1 (0.3)	
LM	17 (2)	11 (3.6)	
PDA from LCx	0 (0)	1 (0.3)	
Graft to LAD	0 (0)	8 (2.6)	
Graft to LCx	0 (0)	28 (9.1)	
Graft to RCA	0 (0)	25 (8.1)	
**Specific lesion location, no. (%)**			
Proximal	413 (47.8)	132 (42.7)	<0.001
Mid	336 (38.9)	86 (27.8)	
Distal	115 (13.3)	20 (6.5)	
**Lesions characteristics, no. (%)**			
Total occlusion	85 (9.8)	136 (44)	<0.001
Eccentric plaque	256 (29.6)	3 (1)	<0.001
**Calcifications**	372 (43.1)	160 (51.8)	<0.001
Mild	92 (10.7)	12 (3.9)	<0.001
Moderate	112 (13)	27 (8.7)	
Severe	168 (19.4)	121 (39.2)	
Diffuse disease	89 (10.3)	36 (11.7)	<0.001
Ostial	73 (8.5)	46 (14.9)	<0.001
Bifurcation lesion	247 (28.6)	123 (39.8)	<0.001
**Medina classification**			
0,0,1	40 (4.6)	0 (0)	<0.001
0,1,0	61 (7.1)	12 (3.9)	
0,1,1	16 (1.9)	4 (1.3)	
1,0,0	30 (3.5)	1 (0.3)	
1,0,1	11 (1.3)	1 (0.3)	
1,1,0	49 (5.7)	17 (5.5)	
1,1,1	40 (4.6)	87 (28.2)	
Flow deceleration	48 (5.6)	0 (0)	<0.001
Pulsatile flow	9 (1)	0 (0)	<0.001
Slow flow	34 (3.9)	0 (0)	<0.001
Collaterals	84 (9.7)	160 (51.8)	<0.001
**Collaterals score**			
1	21 (2.4)	21 (6.8)	<0.001
2	63 (7.3)	139 (45)	
Thrombus	60 (6.9)	0 (0)	<0.001
**Thrombus grade**			
1	4 (0.5)	0 (0)	
2	2 (0.2)	0 (0)	
3	17 (2)	0 (0)	
4	8 (0.9)	0 (0)	
5	29 (3.4)	0 (0)	
Ulcer	19 (2.2)	0 (0)	<0.001
Distal embolization	6 (0.7)	0 (0)	<0.001
**Lesion complexity (ACC/AHA)**			
A	127 (14.7)	7 (2.3)	<0.001
B1	289 (33.5)	27 (8.7)	
B2	318 (36.8)	28 (9.1)	
C	130 (15.1)	171 (55.3)	
LL (mm), median [IQR]	9.2 [6.6, 13.5]	12.7 [9.1, 20.1]	<0.001
RVD (mm), median [IQR]	2.7 [2.2, 3.2]	2.7 [2.2, 3.3]	0.575
Area stenosis (%), median [IQR]	91.8 [83.8, 96.3]	100 [88.1, 100]	<0.001
DS (%), median [IQR]	71.7 [60.1, 81.5]	100 [65.4, 100]	<0.001
Lesion MLD (mm), median [IQR]	0.7 [0.5, 1.1]	0.0 [0, 0.9]	<0.001

**Abbreviations.** ACC/AHA, American College of Cardiology/American Heart Association; CABG, coronary artery bypass grafting; DS, diameter stenosis; IQR, interquartile range; LAD, left anterior descending; LCx, left circumflex; LL, lesion length; LM, left main; MLD, minimal lumen diameter; RCA, right coronary artery; PCI, percutaneous coronary intervention; PDA, posterior-descending artery; PLA, posterior-lateral artery; RVD, reference vessel diameter.

A significantly higher number of total occlusions was reported in patients with prior CABG compared with the non-prior CABG group (44% vs. 9.8%, *P* < 0.001), as well as calcifications (51.8% vs. 43.1%, *P* < 0.001), mostly severe (39.2% vs. 19.4%, *P* < 0.001). Collaterals were more frequently reported in the prior CABG group (51.8% vs. 9.7%, *P* < 0.001). Conversely, a significantly higher percentage of eccentric plaques (29.6% vs. 1%, *P* < 0.001), thrombus detection (6.9% vs. 0%), ulcer (2.2% vs. 0%), and distal embolization (0.7% vs. 0%) were identified in non-prior CABG patients. Most lesions in the non-CABG group were classified as ACC/AHA type B2 (36.8%), while in the prior CABG group, the majority were identified as type C (55.3%).

PCI was performed in 293 non-CABG patients (50.5%) and 37 CABG patients (42.5%). Significant differences were reported in lesion measurements between the two groups, such as lesion length, which was significantly longer in the prior CABG group [12.7 mm, IQR (9.1, 20.1) vs. 9.2 mm, IQR (6.6, 13.5) in the non-CABG group, *P* < 0.001] (*Table 3*).

### Percutaneous coronary intervention procedure

Overall, 49.5% (*n* = 330) of patients underwent PCI, which was performed on 405 lesions in patients without prior CABG and 45 lesions in those with prior CABG (respectively, 46.9% vs. 14.6%, *P* < 0.001). Among patients with prior CABG, PCI was performed in 23 (9.3%) lesions on native vessels and in 22 (36.1%) graft lesions.

Intra-procedural complications were significantly more frequent in patients without prior CABG, although they were negligible. The most frequent complications observed during PCI were flow deceleration, exclusively occurring in 1.4% of treated lesions in the non-CABG group, and slow flow (3.5% of treated lesions in the non-prior CABG group vs. 2.2% of treated lesions in the CABG group, *P* < 0.001).

Post-PCI complications were reported in <1% of patients, except for slow flow, which occurred more frequently in lesions of CABG patients (4.4% vs. 1%, *P* < 0.001) (*Table 4*). The procedure was successful in 96.5% (*n* = 435) of treated lesions. However, there was a small but significant difference in procedural success between lesions in patients without prior CABG and those with prior CABG [96.8% and 95.6%, respectively (*P* < 0.001)].

**Table 4:** PCI characteristics

Lesions, no.	All patients lesions *N* = 1173	Without prior CABG *N* = 864	With prior CABG *N* = 309	*P* value
PCI performed, no. (%)	450 (38.4)	405 (46.9)	45 (14.6)	<0.001
Deceleration during PCI	16 (3.6)	16 (1.4)	0 (0)	<0.001
Pulsatile flow during PCI	3 (0.7)	3 (0.7)	0 (0)	<0.001
No reflow during PCI	5 (1.1)	5 (1.2)	0 (0)	<0.001
Slow flow during PCI	15 (3.3)	14 (3.5)	1 (2.2)	<0.001
Dissection during PCI	7 (1.6)	7 (1.7)	0 (0)	<0.001
Distal embolization during PCI	1 (0.2)	1 (0.3)	0 (0)	<0.001
Abrupt closure during PCI	1 (0.2)	1 (0.3)	0 (0)	<0.001
Thrombus during PCI	4 (0.9)	4 (1)	0 (0)	<0.001
Side branch loss	1 (0.2)	1 (0.3)	0 (0)	<0.001
Stain	1 (0.2)	1 (0.3)	0 (0)	<0.001
Post-PCI deceleration	4 (0.9)	4 (1.0)	0 (0)	<0.001
Post-PCI pulsatile flow	2 (0.4)	2 (0.5)	0 (0)	<0.001
Post-PCI no reflow	3 (0.7)	3 (0.7)	0 (0)	<0.001
Post-PCI slow flow	6 (1.3)	4 (1.0)	2 (4.4)	<0.001
Post-PCI dissection	1 (0.2)	1 (0.3)	0 (0)	<0.001
Post-PCI distal embolization	1 (0.2)	1 (0.3)	0 (0)	<0.001
**Post-PCI angiographic measurements calculated in all the lesions (both native vessels and grafts)**				
Lesions RVD (mm), median [IQR]	2.8 [2.4, 3.2]	2.8 [2.4, 3.2]	3 [2.6, 3.5]	0.064
Area stenosis (%), median [IQR]	24.7 [17.9, 35.8]	23.9 [17.3, 35]	32.7 [21.6, 40.3]	0.006
DS (%), median [IQR]	13.3 [9.5, 20.1]	12.9 [9.3, 19.6]	17.9 [11.2, 22.7]	0.008
MLD (mm), median [IQR]	2.4 [2, 2.8]	2.4 [2, 2.8]	2.5 [2.1, 2.8]	0.539
**Procedure success, no. (%),** calculated on the number of lesions treated with PCI	435 (96.5)	392 (96.8)	43 (95.6)	<0.001
Complete revascularization, no. (%)		188 (64.2)		
Complete revascularization excluding CTO, no. (%)		207 (70.7)		

**Abbreviations.** CABG; coronary artery bypass grafting; CTO, chronic total occlusion; DS, diameter stenosis; IQR, interquartile range; LL, lesion length; MLD, minimal lumen diameter; PCI, percutaneous coronary intervention; RVD, reference vessel diameter.

## Discussion

SENIOR-RITA angiographic sub-study represents the largest subgroup analysis to date in older adults with NSTEMI receiving an invasive strategy, providing important insights into their angiographic characteristics. The main results of the study are as follows:

- Among older adults with NSTEMI allocated to receive an invasive strategy, those who did not receive PCI were more likely to be female, cognitively impaired, and frail.
- Among older adults with NSTEMI undergoing an invasive strategy, obstructive CAD was present in ∼77% of cases, and >10% had prior CABG.
- Among patients with obstructive CAD, a significant proportion had complex lesions including bifurcation disease (31.5%), ostial disease (10.1%), and diffuse disease (22.3%).
- PCI was performed in <50% of patients receiving an invasive strategy. Overall, 64.5% of patients with obstructive CAD underwent PCI, with untreated lesions being more complex and severely calcified and more frequently involving bifurcations.
- Patients with prior CABG had more extensive disease, including a higher prevalence of total occlusions, severe calcifications, longer lesions, and collaterals. PCI was performed in fewer CABG patients compared with non-prior CABG patients.
- Procedural success was high, but success rates were lower in CABG patients compared with non-CABG patients. Intra-procedural complications were slightly more frequent in non-CABG patients, but generally minimal (<1%).

Patients aged ≥75 years constitute 40% of those admitted to the hospital with a diagnosis of NSTEMI.^10^ However, they have been reported to present with more complex CAD and different angiographic characteristics compared with younger patients.^2–4,11^ Despite significant differences in coronary angiographic characteristics between the two groups, guidelines do not discern treatment strategies between them, recommending a holistic approach to individualize interventional and pharmacological treatments after careful evaluation of risks and benefits in older NSTEMI patients.^12^ There is a lack of specific recommendations for older patients due to underrepresentation in clinical research and less frequent referral to undergo invasive procedures.^13–17^ The present findings suggest that clinical characteristics and anatomical complexity may have influenced the decision not to proceed with percutaneous revascularization in a significant proportion of patients, especially in those with prior CABG due to the greater complexity of lesions, deterring operators from encountering periprocedural complications.

The majority of patients (76.8%) had obstructive CAD, while the remaining (23.2%) presented with myocardial infarction with non-obstructive coronary arteries. Though most patients had obstructive CAD, the percentage of those treated with PCI corresponded to <50% of the entire cohort (*n* = 330, 49.5%), a finding comparable to those reported by the recent Association for Acute Cardiovascular Care and the European Association of Percutaneous Cardiovascular Interventions under the umbrella of the EURObservational Research Programme NSTEMI registry and the After Eighty trial.^11,18^ The decision not to proceed with PCI may be explained by patient-level factors such as poorer cognitive function and greater frailty, as indicated by Rockwood and Fried scores. In addition, decision-making may also have been influenced by the presence of very severe CAD in some individuals whom clinicians considered unsuitable for revascularization and in the context of multiple comorbidities.

Consistent with this observation, those who did not undergo PCI had significantly worse angiographic characteristics than those who received PCI, including severe calcifications, bifurcation involvement, and collaterals. In this setting, coronary revascularization might be less beneficial when significant risks outweigh the benefits in older patients.^18,19^ In the SENIOR-RITA trial, 32.4% of patients were frail, and 62.5% had cognitive impairment and a high burden of coexisting conditions. Once coronary anatomy has been defined, optimal medical therapy may be a more appropriate strategy in cases where lesion complexity unfavourably alters the risk–benefit ratio, increasing procedural risk.

Among older patients with myocardial infarction and multivessel CAD, the FIRE trial previously showed that the implementation of a physiology-guided complete revascularization yielded a statistically significant reduction in the risk of the composite of death, reinfarction, stroke, and ischaemia-driven revascularization compared with a culprit-only revascularization strategy.^20^ However, these findings are not transferable to an unselected population of older NSTEMI patients. Patient subgroups without obstructive multivessel CAD were not enrolled in FIRE, as per the study protocol, thus leaving out a segment of the older population in whom other variables besides CAD play a role as mortality predictors, significantly reclassifying risk beyond age.^21,22^ Indeed, where long follow-up is undertaken in older patients, the risk of non-cardiovascular death competes directly with that of cardiovascular death.^23^

Comprehensive geriatric assessment in older adults regarding comorbidities and life expectancy should be performed before proceeding to invasive investigation and revascularization.^24–26^ Apart from clinical characteristics, the pattern of CAD distribution plays a pivotal role in the decision-making process regarding revascularization. Patients with a diffuse pattern of CAD are usually older,^27^ and this specific pattern does not benefit from revascularization in terms of procedural results.^28^

Previous studies excluded patients with a prior history of CABG surgery. In SENIOR-RITA, 13% of patients had previously undergone CABG surgery, and 19.7% of the lesions observed were located on grafts. However, only 42.5% of patients in this subgroup underwent PCI. The remaining portion was evidently deemed unsuitable for further revascularization.

## Study limitations

To date, the present analysis constitutes the most comprehensive study providing robust descriptive angiographic data on a large cohort of older patients presenting with NSTEMI and undergoing an invasive strategy. The descriptive data are derived from a well-balanced population, comprising a substantial proportion of female patients (44.8%), patients with non-obstructive CAD (23.2%), and those with a history of prior surgical revascularization (13.4%),^1^ strengthening the generalizability of our study findings. However, this analysis has several limitations. In the present extended data, we acknowledge that the detailed information is exclusively presented for patients who underwent an invasive strategy. The revascularization data only include details on percutaneous treatment, excluding those individuals who received CABG post-randomization (*n* = 25). Additional limitations related to the angiographic sub-study should be specified, such as non-analysable angiograms due to missing acquisitions resulting from challenging arterial access or unreadable discs. Lesion length analysis did not take into account the considerable number of chronic total occlusions for which this measurement is not available. In addition, the core lab performed QCA, which represents an analysis of each individual lesion, as opposed to quantitative flow ratio, which analyses the entire vessel. The latter is able to identify long lesions across the vessels while also providing a non-invasive pullback pressure gradient value to classify the disease pattern. It should be acknowledged that visual assessment, which was adopted in all SENIOR-RITA procedures to determine the revascularization treatment, typically overestimates lesion severity compared with QCA.^29^

Our manuscript should also be interpreted in the context that the goal of the present manuscript is to provide the descriptive angiographic characteristics of patients in the invasive arm and, therefore, outcomes are not reported according to the complexity of the coronary pathology, any percutaneous treatment, or frailty status. It is not possible from the present analysis to confirm if there are any angiographic subgroups (complex untreated disease) that could have received treatment but did not, and whether it had any impact on the outcome. Developing an angiographic score is beyond the scope of this analysis. Overall, in SENIOR-RITA, the procedural complication rate was <1%. The potential influence of operator and centre-level decision-making in revascularization choices was not formally captured in our study. The decision to treat or not treat complex lesions was left to the discretion of the operator based on patients’ comorbidities to determine real-world practice.

## Conclusion

The SENIOR-RITA angiographic sub-study is the largest subgroup analysis to date conducted in older adults with NSTEMI, providing important insights into the angiographic characteristics and procedural details of patients receiving invasive strategy (Graphical abstract). Though the majority of patients had obstructive CAD, <50% underwent PCI, emphasizing the importance of holistic, patient-centred, individualized care for older adults and the relative safety of PCI in selected patients.

## Clinical perspectives

- What is known?
  The SENIOR-RITA trial found that in older NSTEMI patients, an invasive strategy did not significantly reduce the composite outcome of cardiovascular death or non-fatal MI compared with conservative management. Older adults present with more complex angiographic features, potentially complicating invasive management. Given the clinical heterogeneity of this population, detailed angiographic analysis is necessary to interpret the trial findings and guide individualized treatment.
- What is new?
  The SENIOR-RITA Angio Sub-study provides valuable insights into the coronary anatomy of older NSTEMI adults undergoing invasive strategy. Patients not receiving PCI were more likely to be female, cognitively impaired, frail, and affected by more complex CAD. PCI was performed in fewer than 50% of patients, with untreated lesions more frequently characterized by increased complexity.
- What is next?
  Future research should aim to elucidate the impact of frailty, cognitive impairment, sex, and anatomical complexity on clinical decision-making and outcomes in older NSTEMI adults. Development of tailored risk stratification tools may help optimize treatment strategies in this heterogeneous population.

## Supplementary Material

oeag131_Supplementary_Data

## Data Availability

Data can be made available on request to Sponsor.
