# Supplementary material for: Angiographic characteristics of older patients with non-ST elevation myocardial infarction undergoing invasive strategy: A pre-specified analysis from the SENIOR-RITA trial
Source: Eur Heart J Open. 2026 Aug 20;6(5):oeag131. doi: 10.1093/ehjopen/oeag131 (PMC13583498; doi:10.1093/ehjopen/oeag131)
Supplement: Supplementary file 1 [file oeag131_supplementary_data.docx]

**Angiographic Characteristics of Older Patients with Non-ST Elevation Myocardial Infarction Undergoing Invasive Strategy**

***A Pre-specified Analysis from the SENIOR-RITA Trial***

Table of Contents

[Angiographic Characteristics of Older Patients with Non-ST Elevation Myocardial Infarction Undergoing Invasive Strategy	1](#_Toc235966972)

[A Pre-specified Analysis from the SENIOR-RITA Trial	1](#_Toc235966973)

[Supplemental Table 1. Eligibility criteria	2](#_Toc235966974)

[Supplemental Table 2. Culprit lesions characteristics in non-CABG patients.	3](#_Toc235966975)

[Supplemental Table 3. Characteristics of non-culprit lesions before, during and post-PCI in non-CABG patients.	7](#_Toc235966976)

[Supplemental Table 4: Angiographic characteristics of lesions on native vessels of patients with and without prior CABG	11](#_Toc235966977)

[Supplemental Table 5. Lesion characteristics of non-CABG patients with diseased vessels who did not undergo PCI	17](#_Toc235966978)

[Supplemental Table 6. Angiographic characteristics of CABG patients allocated to the invasive strategy arm.	20](#_Toc235966979)

[Supplemental Table 7. Medical therapy	22](#_Toc235966980)

# **Supplemental Table 1. Eligibility criteria**

**Inclusion criteria - All the following:**

Age ≧75 years

Type 1 NSTEMI during index hospitalisation

**Exclusion criteria - Any of the following:**

Patients presenting with STEMI or unstable angina

Patients with cardiogenic shock

Patients with a known life expectancy of < 1 year

Patients in whom neither the patient nor the consultee are able and willing to provide written informed consent

Previous randomization in the SENIOR RITA trial

Inability to undergo invasive coronary angiography, such as no vascular access site, or absolute contraindication to coronary revascularisation

**Abbreviations.** NSTEMI, non-ST-segment elevation myocardial infarction; STEMI, ST-segment elevation myocardial infarction.

# **Supplemental Table 2. Medical therapy**

| **DISCHARGE MEDICAL THERAPY** | **Invasive Strategy (N=752)** | **Conservative Strategy (N=762)** |
| --- | --- | --- |
| Antiplatelet therapies |  |  |
| Aspirin - no. (%) | 682 (90.7) | 663 (87.0) |
| P2Y_12_ Receptor Antagonist (Total) - no. (%) | 674 (89.6) | 719 (94.4) |
| Clopidogrel - no. (%) | 348 (46.3) | 405 (53.1) |
| Ticagrelor - no. (%) | 322 (42.8) | 313 (41.1) |
| Prasugrel - no. (%) | 4 (0.5) | 1 (0.1) |
| Antiplatelet therapy |  |  |
| None - no. (%) | 20 (2.7) | 8 (1.0) |
| Single - no. (%) | 108 (14.4) | 126 (16.5) |
| Dual - no. (%) | 624 (83.0) | 628 (82.4) |
| Anticoagulant* (Total) - no. (%) | 170 (22.6) | 183 (24.0) |
| Apixaban – no. (%) | 51 (6.8) | 71 (9.3) |
| Rivaroxaban - no. (%) | 44 (5.9) | 38 (5.0) |
| Warfarin - no. (%) | 28 (3.7) | 34 (4.5) |
| Edoxaban - no. (%) | 16 (2.1) | 15 (2.0) |
| Dabigatran - no. (%) | 2 (0.3) | 4 (0.5) |
| Other – no. (%) | 29 (3.9) | 21 (2.8) |
| Triple therapy (Total) - no. (%) | 100 (13.3) | 91 (11.9) |
| Atorvastatin - no. (%) | 595 (79.1) | 608 (79.8) |
| Simvastatin - no. (%) | 40 (5.3) | 43 (5.6) |
| Rosuvastatin - no. (%) | 31 (4.1) | 25 (3.3) |
| Pravastatin – no. (%) | 12 (1.6) | 9 (1.2) |
| Ezetimibe - no. (%) | 4 (0.5) | 3 (0.4) |

# **Supplemental Table 3. Culprit lesions characteristics in non-CABG patients.**

| **Culprit lesions, no.** | 425 |
| --- | --- |
| **Location of culprit lesions, no. (%)** |  |
| LAD | 165 (38.8) |
| LCx | 62 (14.6) |
| RCA | 133 (31.3) |
| Intermediate | 2 (0.5) |
| Diagonal | 11 (2.6) |
| Obtuse marginal | 28 (6.6) |
| RCA postero-lateral | 4 (0.9) |
| PDA | 6 (1.4) |
| LM | 14 (3.3) |
| **Specific culprit location, no. (%)** |  |
| Proximal | 221 (52) |
| Mid | 166 (39.1) |
| Distal | 38 (8.9) |
| **Culprit characteristics, no. (%)** |  |
| Total occlusion | 34 (8.0) |
| Eccentric plaque | 128 (30.1) |
| **Calcifications, no. (%)** | 202 (47.5) |
| Mild | 51 (0.1) |
| Moderate | 58 (13.7) |
| Severe | 93 (21.9) |
| Diffuse disease, no. (%) | 57 (13.4) |
| Ostial, no. (%) | 46 (10.8) |
| Bifurcation lesion, no. (%) | 141 (33.2) |
| **Medina classification, no. (%)**  0,0,1  0,1,0  0,1,1  1,0,0  1,0,1  1,1,0  1,1,1 | 13 (3.1)  41 (9.7)  4 (0.9)  24 (5.7)  3 (0.7)  33 (7.8)  23 (5.4) |
| Flow deceleration, no. (%) | 30 (7.1) |
| Pulsatile flow, no. (%) | 8 (1.9) |
| Slow flow, no. (%) | 21 (4.9) |
| Collaterals, no. (%) | 41 (9.7) |
| **Collaterals score, no. (%)**  1  2 | 13 (3.1)  28 (6.6) |
| Culprit thrombus, no. (%) | 43 (10.1) |
| **Thrombus grade, no. (%)**  1  2  3  4  5 | 2 (0.5)  1 (0.2)  12 (2.8)  7 (1.7)  21 (4.9) |
| Ulcer, no. (%) | 15 (3.5) |
| Distal embolization, no. (%) | 5 (1.2) |
| **Lesion complexity (ACC/AHA), no. (%)** |  |
| A  B1  B2  C | 34 (8)  130 (30.6)  189 (44.5)  72 (16.9) |
| Lesion stain, no. (%) | 7 (1.7) |
| LL (mm), median [IQR] | 10.1 [7.0 -14.6] |
| RVD (mm), median [IQR] | 2.8 [2.3 - 3.4] |
| Area stenosis (%), median [IQR] | 93 [86.9 - 97.3] |
| DS (%), median [IQR] | 73.8 [64 - 84.4] |
| MLD (mm), median [IQR] | 0.7 [0.4 - 1.0] |
| **PCI performed, no. (%)** | 292 (68.7) |
| Deceleration during PCI, no. (%) | 12 (4.1) |
| Pulsatile flow during PCI, no. (%) | 2 (0.7) |
| No reflow during PCI, no. (%) | 4 (1.4) |
| Slow flow during PCI, no. (%) | 12 (4.1) |
| Dissection during PCI, no. (%) | 7 (2.4) |
| Distal embolization during PCI, no. (%) | 1 (0.3) |
| Abrupt closure during PCI, no. (%) | 0 (0) |
| Thrombus during PCI, no. (%) | 2 (0.7) |
| Perforation during PCI, no. (%) | 0 (0) |
| Loss of side branch during PCI, no. (%) | 1 (0.3) |
| Stain during PCI, no. (%) | 1 (0.3) |
| **Procedure success, no (%)** | 285 (97.6) |
| Post-PCI deceleration | 4 (1.4) |
| Pulsatile flow | 1 (0.3) |
| No reflow | 1 (0.3) |
| Slow flow | 4 (1.4) |
| Dissection | 1 (0.3) |
| Distal embolization | 1 (0.3) |
| Abrupt closure | 0 (0) |
| Thrombus | 0 (0) |
| Post-PCI perforation | 0 (0) |
| Loss of side branch | 0 (0) |
| Post-PCI stain | 0 (0) |
| Post-PCI LL (mm), median [IQR] | 3.1 [2.2, 4.6] |
| Post-PCI RVD (mm), median [IQR] | 2.9 [2.4, 3.3] |
| Post-PCI area stenosis (%), median [IQR] | 24.3 [18, 35.5] |
| Post-PCI DS (%), median [IQR] | 13.2 [9.5, 19.8] |
| Post-PCI MLD (mm), median [IQR] | 2.4 [2.0, 2.9] |

**Abbreviations.** ACC/AHA, American College of Cardiology/American Heart Association; DS, diameter stenosis; IQR, interquartile range; LAD, left anterior descending; LCx, left circumflex; LL, lesion length; LM, left main; MLD, minimal lumen diameter; PCI, percutaneous coronary intervention; PDA, postero-descending artery; RCA, right coronary artery; RVD, reference vessel diameter.

# **Supplemental Table 4. Characteristics of non-culprit lesions before, during and post-PCI in non-CABG patients.**

| **Non-culprit lesions, no.** | 439 |
| --- | --- |
| **Non-culprit lesion location, no. (%)** |  |
| **LAD** | 123 (28) |
| **LCx** | 91 (20.7) |
| RCA | 131 (29.8) |
| Intermediate | 10 (2.3) |
| Diagonal | 32 (7.3) |
| Obtuse marginal | 31 (7.1) |
| RCA postero-lateral | 12 (2.7) |
| PDA | 6 (1.4) |
| LM | 3 (0.7) |
| **Specific location, no. (%)** |  |
| Proximal  Mid  Distal | 192 (43.7)  170 (38.7)  77 (17.5) |
| Occluded, no. (%) | 51 (11.6) |
| Eccentric, no. (%) | 128 (29.2) |
| Calcifications, no. (%) | 170 (38.7) |
| **Calcification score, no. (%)** |  |
| Mild | 41 (9.3) |
| Moderate | 54 (12.3) |
| Severe | 75 (17.1) |
| Diffuse disease, no. (%) | 32 (7.3) |
| Ostial, no. (%) | 27 (6.2) |
| Bifurcation, no. (%) | 106 (24.2) |
| **Medina, no. (%)** |  |
| 0,0,1  0,1,0  0,1,1  1,0,0  1,0,1  1,1,0  1,1,1 | 27 (6.2)  20 (4.6)  12 (2.7)  6 (1.4)  8 (1.8)  16 (3.7)  17 (3.9) |
| Flow deceleration, no. (%) | 18 (4.1) |
| Pulsatile flow, no. (%) | 1 (0.2) |
| Slow flow, no. (%) | 13 (3) |
| Collaterals, no. (%) | 43 (9.8) |
| **Collaterals score, no. (%)** |  |
| 1  2 | 8 (1.8)  35 (8) |
| Thrombus, no. (%) | 17 (3.9) |
| **Thrombus score, no. (%)** |  |
| 1  2  3  4  5 | 2 (0.5)  1 (0.2)  5 (1.1)  1 (0.2)  8 (1.8) |
| Ulcer, no. (%) | 4 (0.9) |
| Distal embolization, no. (%) | 1 (0.2) |
| **Lesion complexity (ACC/AHA), no. (%)** |  |
| A  B1  B2  C | 93 (21.2)  159 (36.2)  129 (29.4)  58 (13.2) |
| Stain, no. (%) | 7 (1.6) |
| LL pre-PCI (mm), median [IQR] | 8.5 [6.2, 12.1] |
| RVD (mm), median [IQR] | 2.5 [2.1, 3.1] |
| Area stenosis (%), median [IQR] | 89.4 [81.7, 95.2] |
| DS (%), median [IQR] | 68.2 [57.6, 79.0] |
| MLD (mm), median [IQR] | 0.8 [0.5, 1.1] |
| **PCI performed, no. (%)** | 113 (25.7) |
| Deceleration during PCI | 4 (3.5) |
| Pulsatile flow during PCI | 1 (0.9) |
| No reflow during PCI | 1 (0.9) |
| Slow flow during PCI | 2 (1.8) |
| Dissection during PCI | 0 (0) |
| Distal embolization during PCI | 0 (0) |
| Abrupt closure during PCI | 1 (0.9) |
| Thrombus during PCI | 2 (1.8) |
| Perforation | 0 (0) |
| Side branch loss | 0 (0) |
| Stain | 0 (0) |
| **Procedure success, no. (%)** | 108 (95.6) |
| Post-PCI deceleration, no. (%) | 0 (0) |
| Post-PCI pulsatile flow, no. (%) | 1 (0.9) |
| No reflow post-PCI, no. (%) | 1 (0.9) |
| Post-PCI slow flow, no. (%) | 0 (0) |
| Post-PCI dissection, no. (%) | 0 (0) |
| Post-PCI distal embolization, no. (%) | 0 (0) |
| Post-PCI abrupt closure, no. (%) | 0 (0) |
| Post-PCI thrombus, no. (%) | 0 (0) |
| Perforation, no. (%) | 0 (0) |
| Side branch loss, no. (%) | 0 (0) |
| Stain, no. (%) | 0 (0) |
| Post-PCI LL (mm), median [IQR] | 3.2 [2.3, 4.5] |
| Post-PCI RVD (mm), median [IQR] | 2.7 [2.4, 3] |
| Post-PCI area stenosis (%), median [IQR] | 22.4 [16.3, 34.3] |
| Post-PCI DS (%), median [IQR] | 12 [8.8, 19.2] |
| Post-PCI MLD (mm), median [IQR] | 2.3 [2, 2.6] |

**Abbreviations.** ACC/AHA, American College of Cardiology/American Heart Association; DS, diameter stenosis; IQR, interquartile range; LAD, left anterior descending; LCx, left circumflex; LL, lesion length; LM, left main; MLD, minimal lumen diameter; PCI, percutaneous coronary intervention; PDA, postero-descending artery; RCA, right coronary artery; RVD, reference vessel diameter.

# **Supplemental Table 5: Angiographic characteristics of lesions on native vessels of patients with and without prior CABG**

| **Angiographic characteristics of lesions on native vessels** | **Non-prior CABG patients**  **N= 580** | **Prior CABG patients**  **N=87** | **P value** |
| --- | --- | --- | --- |
| **Total number of lesions on native vessels, no. (%)** | 864 | 248 |  |
| 0 | 155 (26.7) | 0 (0) | <0.001 |
| 1 | 174 (30.0) | 3 (1.2) |  |
| 2 | 131 (22.6) | 17 (6.9) |  |
| 3 | 81 (14.0) | 58 (23.4) |  |
| 4 | 20 (3.5) | 8 (3.2) |  |
| 5 | 10 (1.7) | 1 (0.4) |  |
| 6 | 8 (1.4) | 0 (0) |  |
| 7 | 1 (0.2) | 0 (0) |  |
| Myocardial bridge, no. (%) | 4 (0.7) | 0 (0) | 0.656 |
| **Pre-PCI quantitative analysis** |  |  |  |
| **Pre-PCI TIMI flow grade, no. (%)** |  |  |  |
| **LAD** |  |  |  |
| 0 | 16 (2.8) | 56 (64.4) | <0.001 |
| 1 | 4 (0.7) | 0 (0) |  |
| 2 | 14 (2.4) | 0 (0) |  |
| 3 | 539 (92.9) | 20 (23) |  |
| NA | 7 (1.2) | 11 (12.6) |  |
| **LCx** |  |  |  |
| 0 | 7 (1.2) | 34 (39.1) | <0.001 |
| 1 | 1 (0.2) | 0 (0) |  |
| 2 | 3 (0.5) | 0 (0) |  |
| 3 | 562 (96.9) | 48 (55.2) |  |
| NA | 7 (1.2) | 5 (5.8) |  |
| **RCA** |  |  |  |
| 0 | 32 (5.5) | 43 (49.4) | <0.001 |
| 1 | 13 (2.2) | 0 (0) |  |
| 2 | 9 (1.6) | 2 (2.3) |  |
| 3 | 510 (87.9) | 36 (41.4) |  |
| NA | 16 (2.8) | 6 (6.9) |  |
| **Pre-PCI TIMI frame count, median [IQR]** |  |  |  |
| LAD | 21 [16-27] | 17 [13.5, 19.5] | 0.024 |
| LCx | 16 [11-20] | 15 [11.8, 19.2] | 0.873 |
| RCA | 14 [10-20] | 16.5 [9.5, 21.3] | 0.630 |
| **Pre-PCI TIMI myocardial perfusion grade, no. (%)** |  |  |  |
| **LAD** |  |  |  |
| 0 | 7 (1.2) | 0 (0) | <0.001 |
| 1 | 4 (0.7) | 1 (1.2) |  |
| 2 | 20 (3.5) | 15 (17.2) |  |
| 3 | 539 (92.9) | 59 (67.8) |  |
| NA | 10 (1.7) | 0 (0) |  |
| **LCx** |  |  |  |
| 0 | 2 (0.4) | 0 (0) | <0.001 |
| 1 | 4 (0.7) | 0 (0) |  |
| 2 | 8 (1.4) | 0 (0) |  |
| 3 | 558 (96.2) | 70 (80.5) |  |
| NA | 8 (1.4) | 0 (0) |  |
| **RCA** |  |  | <0.001 |
| 0 | 24 (4.1) | 0 (0) |  |
| 1 | 16 (2.8) | 4 (4.6) |  |
| 2 | 16 (2.8) | 18 (20.7) |  |
| 3 | 504 (86.9) | 55 (63.2) |  |
| NA | 19 (3.3) | 0 (0) |  |
| **Patients receiving PCI, no. (%)** | 293 (50.5) | 37 (42.5) |  |
| **Post-PCI quantitative analysis** |  |  |  |
| **TIMI flow grade, no. (%)** |  |  |  |
| **LAD** |  |  |  |
| 0 | 1 (0.3) | 0 (0) | <0.001 |
| 1 | 0 (0) | 0 (0) |  |
| 2 | 1 (0.3) | 0 (0) |  |
| 3 | 147 (50.2) | 2 (5.4) |  |
| NA | 3 (1) | 1 (2.7) |  |
| PCI not done | 428 | 84 |  |
| **LCx artery** |  |  |  |
| 0 | 0 (0) | 0 (0) | <0.001 |
| 1 | 0 (0) | 0 (0) |  |
| 2 | 1 (0.3) | 1 (2.7) |  |
| 3 | 82 (28) | 10 (27) |  |
| NA | 1 (0.3) | 1 (2.7) |  |
| PCI not done | 496 | 75 |  |
| **RCA** |  |  |  |
| 0 | 1 (0.3) | 0 (0) | <0.001 |
| 1 | 1 (0.3) | 0 (0) |  |
| 2 | 4 (1.4) | 0 (0) |  |
| 3 | 99 (33.8) | 7 (18.9) |  |
| PCI not done | 475 | 80 |  |
| **Post-PCI TIMI frame count, median [IQR]** |  |  |  |
| LAD | 15 [10.3, 20.6] | 18 [18, 18] | 0.508 |
| LCx | 12.5 [8, 16.8] | 13 [10, 16] | 0.633 |
| RCA | 9 [6,15] | 14.5 [11, 17.3] | 0.100 |
| **TIMI myocardial perfusion grade, no. (%)** |  |  |  |
| **LAD** |  |  |  |
| 0 | 0 (0) | 0 (0) | <0.001 |
| 1 | 0 (0) | 0 (0) |  |
| 2 | 1 (0.3) | 0 (0) |  |
| 3 | 148 (50.5) | 2 (5.4) |  |
| NA | 3 (1) | 1 (2.7) |  |
| **LCx** |  |  |  |
| 0 | 0 (0) | 0 (0) | <0.001 |
| 1 | 0 (0) | 0 (0) |  |
| 1 | 0 (0) | 0 (0) |  |
| 3 | 83 (28.3) | 11 (29.7) |  |
| NA | 1 (0.3) | 1 (2.7) |  |
| **RCA** |  |  |  |
| 0 | 0 (0) | 0 (0) | <0.001 |
| 1 | 3 (1) | 0 (0) |  |
| 2 | 4 (1.4) | 0 (0) |  |
| 3 | 98 (33.5) | 7 (18.9) |  |
| NA | 0 (0) | 1 (2.7) |  |

**Abbreviations.** CABG, coronary artery bypass grafting; IQR, interquartile range; LAD, left anterior descending; LCx, left circumflex; NA, not available; RCA, right coronary artery; PCI, percutaneous coronary intervention; TIMI, Thrombolysis in Myocardial Infarction.

# **Supplemental Table 6. Lesion characteristics of non-CABG patients with diseased vessels who did not undergo PCI**

| **Total number of patients with diseased vessels not treated with PCI, no. (%)** | 132 |
| --- | --- |
| Number of lesions, no. | 302 |
| **Lesion location, no. (%)** |  |
| LAD | 84 (27.8) |
| LCx | 62 (20.5) |
| RCA | 102 (33.8) |
| Intermediate | 5 (1.7) |
| Diagonal | 16 (5.3) |
| Obtuse marginal | 19 (6.3) |
| RCA postero-lateral | 7 (2.3) |
| PDA | 1 (0.3) |
| LM | 6 (2.0) |
| **Specific location, no. (%)**  Proximal  Mid  Distal | 148 (49.0)  109 (36.1)  45 (14.9) |
| Occluded, no. (%) | 85 (28.2) |
| Eccentric, no. (%) | 87 (28.8) |
| Calcifications, no. (%) | 145 (48.0) |
| **Calcification score, no. (%)**  Mild  Mod  Severe | 26 (8.6)  56 (18.5)  63 (20.9) |
| Diffuse disease, no. (%) | 34 (11.3) |
| Ostial, no. (%) | 33 (10.9) |
| Bifurcation, no. (%) | 87 (28.8) |
| **Medina, no. (%)**  0,0,1  0,1,0  0,1,1  1,0,0  1,0,1  1,1,0  1,1,1 | 19 (6.3)  25 (8.3)  3 (1.0)  8 (2.7)  2 (0.7)  17 (5.6)  13 (4.3) |
| Flow deceleration, no. (%) | 17 (5.6) |
| Pulsatile flow, no. (%) | 3 (1.0) |
| Slow flow, no. (%) | 10 (3.3) |
| Collaterals, no. (%) | 44 (14.6) |
| **Collaterals score, no. (%)**  1  2 | 9 (3.0)  35 (11.6) |
| Thrombus, no. (%) | 18 (6.0) |
| **Thrombus score, no. (%)**  1  2  3  4  5 | 2 (0.7)  0 (0)  4 (1.3)  1 (0.3)  11 (3.6) |
| Ulcer, no. (%) | 4 (1.3) |
| Distal embolization, no. (%) | 1 (0.3) |
| **Lesion complexity (ACC/AHA), no. (%)**  A  B1  B2  C | 38 (12.6)  91 (30.1)  115 (38.1)  58 (19.2) |
| Stain, no. (%) | 8 (2.7) |
| LL (mm), median [IQR] | 8.8 [6.3, 14] |
| Lesion RVD (mm), median [IQR] | 2.7 [2.2, 3.4] |
| Area stenosis (%), median [IQR] | 92.3 [83.9, 96.7] |
| DS (%), median [IQR] | 72.5 [60.0, 82.7] |
| MLD (mm), median [IQR] | 0.7 [0.4, 1.1] |

**Abbreviations.** PCI, percutaneous coronary intervention; LAD, left anterior descending; LCx, left circumflex; RCA, right coronary artery; PDA, posterior-descending artery; LM, left main; ACC/AHA, American College of Cardiology/American Heart Association; LL, lesion length; RVD, reference vessel diameter; DS, diameter stenosis; MLD, minimal lumen diameter; IQR, interquartile range; mm, millimetres.

# **Supplemental Table 7. Angiographic characteristics of CABG patients allocated to the invasive strategy arm.**

| **CABG patients, no.** | 87 |
| --- | --- |
| **Total number of diseased native vessels, no.** | 232 |
| **Number of lesions on native vessels, no.** | 248 |
| **PCI performed, no. (%)** | 23 (9.3) |
| Deceleration during PCI | 0 (0) |
| Pulsatile flow during PCI | 0 (0) |
| No reflow during PCI | 0 (0) |
| Slow flow during PCI | 1 (4.4) |
| Dissection during PCI | 0 (0) |
| Distal embolization during PCI | 0 (0) |
| Abrupt closure during PCI | 0 (0) |
| Thrombus during PCI | 0 (0) |
| Perforation | 0 (0) |
| Side branch loss | 0 (0) |
| Stain | 0 (0) |
| **Procedure success, no. (%)** | 22 (95.7) |
| **Not available data, no. (%)** | 1 (4.4) |
| Post-PCI deceleration | 0 (0) |
| Post-PCI pulsatile flow | 0 (0) |
| Post-PCI no reflow | 0 (0) |
| Post-PCI slow flow | 1 (0.0) |
| Post-PCI dissection | 0 (0) |
| Post-PCI distal embolization | 0 (0) |
| Post-PCI abrupt closure | 0 (0) |
| Post-PCI thrombus | 0 (0) |
| Perforation | 0 (0) |
| Side branch loss | 0 (0) |
| Stain | 0 (0) |
| Post-PCI LL (mm), median [IQR] | 3.5 [2.6, 5.6] |
| Post-PCI RVD (mm), mean (SD) | 3.2 (0.6) |
| Post-PCI area stenosis (%), mean (SD) | 32.7 (12.4) |
| Post-PCI DS (%), mean (SD) | 18.3 (7.7) |
| Post-PCI MLD (mm), mean (SD) | 2.6 (0.6) |
| **Total number of grafts, no. (%)** | 175 |
| LAD | 71 (40.6) |
| LCx | 57 (32.6) |
| RCA | 46 (26.3) |
| Intermediate | 1 (0.6) |
| **Total numbers of graft lesions, no.** | 61 |
| Occluded grafts, no. (%) | 34 (19.4) |
| PCI performed , no. (%) | 22 (36.1) |
| **Pre-PCI measurements on graft lesions** |  |
| Pre-PCI LL (mm), median [IQR] | 11.2 [9.00, 16.2] |
| Lesion RVD (mm), mean (SD) | 3.5 (0.9) |
| Pre-PCI area stenosis (%), median [IQR] | 100 [86.6, 100] |
| Pre-PCI DS (%), median [IQR] | 100 [63.5, 100] |
| Pre-PCI MLD (mm), median [IQR] | 0.00 [0, 1.2] |
| **Post-PCI measurements on graft lesions** |  |
| Post-PCI LL (mm), median [IQR] | 4.3 [3.6, 6.4] |
| Post-PCI RVD (mm), median [IQR] | 2.91 [2.6, 3.4] |
| Post-PCI area stenosis (%), median [IQR] | 30.4 [21.1, 39.5] |
| Post-PCI DS (%), median [IQR] | 16.6 [11.2, 22.2] |
| Post-PCI MLD (mm), mean (SD) | 2.4 (0.6) |
| **Total number of lesions (on grafts and native vessels), no.** | 309 |
| **PCI performed (on both graft and native vessel lesions), no. (%)** | 45 (14.6) |

**Abbreviations.** PCI, percutaneous coronary intervention; LAD, left anterior descending; LCx, left circumflex; RCA, right coronary artery; LL, lesion length; RVD, reference vessel diameter; DS, diameter stenosis; MLD, minimal lumen diameter; IQR, interquartile range; mm, millimetre
